# Cannabinoid Ligand‐Mediated Glycogen Depletion in Astrocytes Is Associated With Increased Intracellular Calcium, Energy Metabolism, and Membrane Dynamics

**DOI:** 10.1111/jnc.70332

**Published:** 2025-12-28

**Authors:** Katja Fink, Robert Zorec, Marko Kreft

**Affiliations:** ^1^ Laboratory of Neuroendocrinology‐Molecular Cell Physiology, Institute of Pathophysiology, Faculty of Medicine University of Ljubljana Ljubljana Slovenia; ^2^ Celica Biomedical Ljubljana Slovenia; ^3^ Department of Biology, Biotechnical Faculty University of Ljubljana Ljubljana Slovenia

**Keywords:** astrocytes, calcium signaling, cannabinoid receptors, exocytosis, glucose metabolism, lactate

## Abstract

Astrocytes orchestrate brain energy metabolism and respond to endocannabinoids via cannabinoid receptor type 1 (CB1R), whereas the contribution of CB2R remains uncertain. We combined live‐cell Förster resonance energy transfer sensors for D‐glucose and L‐lactate, intracellular Ca^2+^ imaging, glycogen assays, and whole‐cell patch‐clamp capacitance measurements to define how cannabinoid ligands shape astrocyte physiology in primary rat cultures. The CB1‐selective agonist ACEA triggered rapid, transient elevations in [Ca^2+^]ᵢ and metabolic readouts, whereas the CB2‐biased ligands AM1241 and Gp1a produced sustained metabolic effects, including prolonged increases in intracellular D‐glucose and L‐lactate. AM1241 additionally evoked glycogen depletion. Ligand applications also increased membrane capacitance, consistent with enhanced exocytotic activity and altered membrane dynamics. CB1 immunoreactivity predominated over a weak CB2‐like signal, and RT‐qPCR detected Cnr1 but not Cnr2 transcripts under our conditions. Accordingly, we interpret AM1241/Gp1a actions as ligand‐evoked effects that are predominantly CB1‐linked (and/or off‐target at the concentrations used). Together, these results show that cannabinoid ligands robustly remodel astrocytic energy metabolism and membrane behavior chiefly through CB1‐associated pathways, highlighting a functional axis between cannabinoid signaling, Ca^2+^ mobilization, glycogen remodeling, and exocytosis in astrocytes.

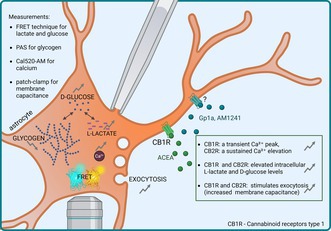

## Graphical abstract

We examined how cannabinoid receptor activation shapes astrocytic energy metabolism and membrane dynamics. Live‐cell Förster resonance energy transfer (FRET) microscopy showed that stimulation with the CB1‐selective agonist ACEA or the CB2‐biased ligands AM1241 and Gp1a increased intracellular D‐glucose ([D‐glucose]ᵢ) and L‐lactate ([L‐lactate]ᵢ) in isolated rat astrocytes. AM1241‐evoked stimulation also promoted glycogen depletion, consistent with a shift toward sustained glucose utilization. Intracellular calcium ([Ca^2+^]ᵢ) measurements revealed distinct temporal patterns for ACEA, AM1241, and Gp1a, indicating ligand‐specific Ca^2+^ signaling profiles. Because Ca^2+^ is a key trigger for exocytosis, we performed whole‐cell patch‐clamp recordings to monitor membrane capacitance as a proxy for exocytotic activity. Cannabinoid ligand application significantly increased astrocyte membrane capacitance, supporting a role for cannabinoid signaling in modulating membrane dynamics. Importantly, although AM1241 and Gp1a are commonly used as CB2 agonists, Cnr2 transcripts were undetectable in our cultures, so their metabolic and capacitance effects are interpreted as ligand‐evoked responses that are most likely CB1‐linked and/or off‐target under our conditions.

**Abbreviations**
ACEA: arachidonyl‐2′‐chloroethylamide
[Ca^2+^]_i_
: intracellular calcium concentration
CB1R: cannabinoid receptor type 1
CB2R: cannabinoid receptor type 2
*C*_m_
: membrane capacitance
[D‐glucose]_i_
: intracellular D‐glucose concentration
eCB: endocannabinoid
eCS: endocannabinoid system
FRET: Förster resonance energy transfer
GPCR: G‐protein‐coupled receptor
HEPES: 4‐(2‐hydroxyethyl)‐1‐piperazineethanesulfonic acid
I_DC_
: direct current
Kruskal–Wallis ANOVA: Kruskal–Wallis one‐way analysis of variance on ranks
[L‐lactate]_i_
: intracellular L‐lactate concentration
PAS: periodic acid‐Schiff
PBS: phosphate‐buffered saline

## 1 Introduction

In the brain, the endocannabinoid system (eCS) is primarily manifested as retrograde control over neurotransmitter release from presynaptic neurons (Wilson and Nicoll 2001; Lu and Mackie 2021). The eCS comprises endocannabinoids (eCBs), enzymes involved in their synthesis and degradation, and two well‐characterized receptors, cannabinoid receptor type 1 (CB1R) and cannabinoid receptor type 2 (CB2R) (Boczek and Zylinska 2021). The eCB signaling system regulates numerous biological processes such as pain perception, learning and memory, appetite modulation, anxiety regulation, and cognitive function (Navarrete et al. 2014; Skaper and Di Marzo 2012; Puighermanal et al. 2012). Astrocytes, integral components of the tripartite synapse, play a crucial role in brain signaling, behavior and metabolism (Araque et al. 1999). Notably, astrocytes are equipped with active eCB receptors on their membrane (Navarrete et al. 2014; Kofalvi et al. 2016), suggesting their involvement, alongside neurons, in the network of eCB signaling. Activation of CB1 receptors can induce the release of gliotransmitters from astrocytes to regulate synaptic plasticity (Covelo et al. 2021; Mariani et al. 2025). Due to their electrically nonexcitable nature, astrocytes primarily utilize calcium (Ca^2+^) signaling as an essential mechanism for intercellular communication and metabolic regulation within the context of the eCB system (Wang et al. 2009).

Cannabinoid receptors engage with several G‐proteins, eliciting a spectrum of cellular responses. CB1R and CB2R are primarily linked to G_i/o_ proteins, initiating the canonical signaling cascade characterized by the inhibition of adenylyl cyclase and subsequent reduction in cAMP production (Howlett et al. 2002; Lograno and Romano 2004; Navarrete and Araque 2008). G_i/o_ activation also modulates intracellular Ca^2+^ release via phospholipase C with the βγ subunit (Exton 1996). Furthermore, G_i/o_ inhibits voltage‐gated N‐ and P−/Q‐type Ca^2+^ channels (Twitchell et al. 1997) while activating inward rectifying potassium channels, as observed in CB1R activation (McAllister and Glass 2002). Conversely, an increase in CB1R‐induced Ca^2+^ may occur through activation of L‐type Ca^2+^ channels via protein kinase A or protein kinase C phosphorylation (Rubovitch et al. 2002). This effect might be mediated by CB1R G_q/11_‐mediated protein kinase C (Lauckner et al. 2005) or G_s_‐mediated protein kinase A activity (Kamp and Hell 2000). The functional G_q/11_‐mediated CB1R pathway for Ca^2+^ release from the endoplasmic reticulum has been investigated in rat hippocampal astrocytes (Navarrete and Araque 2008). Notably, this action can be ligand‐selective because not all CB1R agonists activate G_q_ (Lauckner et al. 2005). Although the connection between CB2R and G_q/11_ proteins has been explored (Brailoiu et al. 2014), specific research elucidating this connection is limited or inconclusive (Chen et al. 2010). A mathematical model (Yang et al. 2023) and cAMP data (Glass and Felder 1997) support the hypothesis that under particular circumstances, such as co‐activation of dopamine D2 receptors with precoupling of G_i/o_, CB1R action switches to G_s_, resulting in enhanced cAMP production. Studies on expressed CB1R or CB2R across various cell types have demonstrated that CB1R can indeed couple with G_s_ and induce cAMP accumulation (Bonhaus et al. 1998; Chen et al. 2010), whereas CB2R typically do not exhibit this association (Chen et al. 2010). These findings underscore receptor‐specific disparities in G‐protein coupling and subsequent signaling pathways.

Although the presence of CB1R in the central nervous system is well established (Duarte et al. 2012; Gutierrez‐Rodriguez et al. 2018; Han et al. 2012; Navarrete and Araque 2008), the expression of CB2R is still a subject of debate and studies have reported conflicting findings. Some investigations have failed to detect CB2R expression in the central nervous system (Bouaboula et al. 1995; Viscomi et al. 2009; Núñez et al. 2004; Palazuelos et al. 2009; Dowie et al. 2014), whereas others have reported its presence (Van Sickle et al. 2005; Gong et al. 2006; Benito et al. 2003), including in glial cells, particularly astrocytes (Gong et al. 2006; Jia et al. 2020), including human astrocytes (Sheng et al. 2005), and isolated gliosomes (Bari et al. 2011). Furthermore, CB2R expression in astrocytes may be associated with neurodegenerative diseases (Rodríguez‐Cueto et al. 2014; Sánchez et al. 2001; Sagredo et al. 2009; Ellert‐Miklaszewska et al. 2007; Benito et al. 2007; Jia et al. 2020).

**FIGURE 1:**
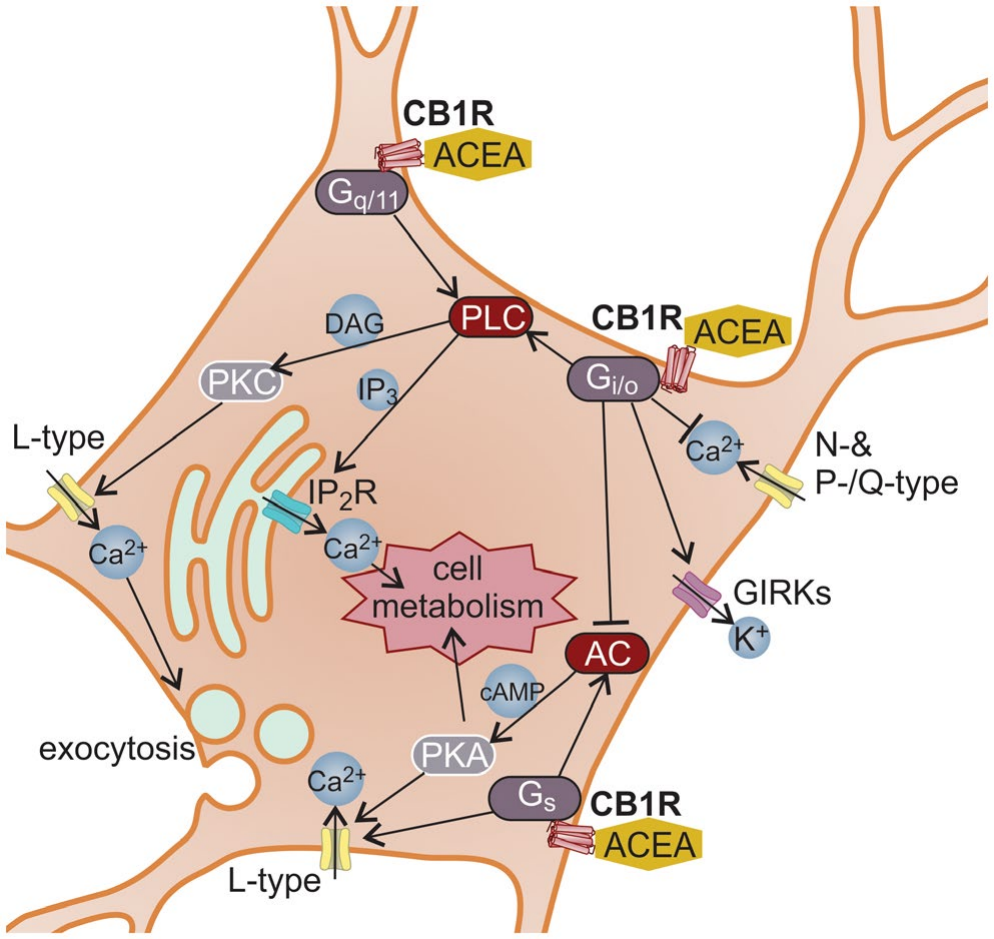
Metabolic pathways activated via G‐protein‐coupled CB1R (ACEA agonist). Flat‐headed arrows indicate inhibition of signaling pathways, while pointed arrows represent stimulation and the direction of signal transduction. AC, adenylyl cyclase; PKA, protein kinase A; PKC, protein kinase C; PLC, phospholipase C.

Energy metabolism is a key component of astrocytic physiology because astrocytes provide essential support to neurons through aerobic glycolysis (Pellerin and Magistretti 2012). Cannabinoid exposure alters glucose oxidation and glycogen content in astrocytes (Sánchez et al. 1998). Activation of CB1R in astrocytes was shown to produce time‐dependent changes in astrocytic lactate levels (Fernández‐Moncada et al. 2024), a response that can proceed either through G_s_‐mediated stimulation of adenylyl‐cyclase (Glass and Felder 1997; Chen et al. 2010) or via G_i/o_ or G_q/11_‐mediated intracellular Ca^2+^ release, as depicted schematically in Figure 1.

In this study, we confirmed expression of CB1R in cultured rat astrocytes, whereas CB2R showed only weak, ambiguous immunolabeling. We used receptor‐selective or CB2‐biased ligands (arachidonyl‐2′‐chloroethylamide [ACEA] for CB1R and Gp1a and (rac)‐AM1241 previously used as CB2R agonists) to probe cannabinoid receptor–dependent responses.

We investigated whether Ca^2+^ plays a role in CBR signaling because this could provide a crucial link to D‐glucose and L‐lactate regulation. Our previous studies (Horvat et al. 2021) demonstrated that increased intracellular Ca^2+^ significantly enhances the levels of these metabolites in astrocytes, surpassing the moderate influence of cAMP. In astrocytes, elevations of intracellular Ca^2+^ are the principal trigger for exocytosis and the consequent release of transmitters, a process termed gliotransmission (Kreft et al. 2004). Cannabinoid receptors, particularly CB1R, impact these Ca^2+^ transients, creating a bidirectional communication pathway between neurons and glia via vesicular gliotransmitter release (Kofuji and Araque 2021a; Eraso‐Pichot et al. 2023). Because Ca^2+^ is central to this mechanism, our study specifically investigates how eCB signaling shapes astrocytic membrane dynamics.

According to recent large‐scale transcriptomic work, Cnr2 (CB2) mRNA is very low across mouse brain regions, with minimal expression in astrocyte‐enriched fractions (Grabon et al. 2024). We therefore treat responses to AM1241 and Gp1a as ligand‐evoked effects that are most likely CB1‐linked and/or off‐target under our conditions. Against this molecular background, the present study asks how cannabinoid ligands, probed with the CB1‐selective agonist ACEA and the CB2‐biased ligands AM1241 and Gp1a, shape astrocytic metabolism and membrane dynamics.

## 2 Materials and Methods

### 2.1 Cell Culture

Experimental procedures involving animals were conducted according to the International Guiding Principles for Biomedical Research Involving Animals developed by the Council for International Organizations of Medical Sciences, and Animal Protection Act (Official Gazette of the RS, No. 38/13) and official consolidated text (21/18, 92/20, 159/21). The experimental protocol was approved by the Administration for Food Safety, Veterinary Sector and Plant Protection (Republic of Slovenia, Ministry of Agriculture, Forestry and Food, Dunajska cesta 22, 1000 Ljubljana), permit numbers U34401‐30/2021/8, U34401‐27/2020/6, U34401‐26/2020/4. All experiments were conducted on rat astrocytes isolated from at least two different animals.

Primary astrocyte cultures were established from the cerebral cortices of male and female neonatal Wistar Han outbred rats (RccHan:WIST; RRID:RGD_149735906; Inotiv, The Netherlands, both sexes) (postnatal days 2–3), in total 80 animals, which were sacrificed by rapid decapitation without prior anesthesia. Astrocyte cultures generated from these animals were aliquoted and distributed to several projects within our laboratory. This procedure was conducted following EU Directive 2010/63/EU on the protection of animals used for scientific purposes and followed the method previously described (Schwartz and Wilson 1992). On reaching confluency, achieved by overnight shaking at 225 rpm and repeated three times at room temperature, astrocyte cultures were purified of other brain cells. The culture medium consisted of high‐glucose Dulbecco's modified Eagle's medium (DMEM; Sigma‐Aldrich, St. Louis, MO, USA, Cat. No. D‐5671) supplemented with 10% fetal bovine serum (Sigma‐Aldrich, St. Louis, MO, USA, Cat. No. F‐7524), 1 mM sodium pyruvate (Sigma‐Aldrich, St. Louis, MO, USA, Cat. No. S‐8636), 2 mM L ‐glutamine (Sigma‐Aldrich, St. Louis, MO, USA, Cat. No. G‐3126), and 25 μg/mL penicillin–streptomycin (Sigma‐Aldrich, St. Louis, MO, USA, Cat. No. P‐0781), maintained in an atmosphere of 95% humidified air and 5% CO_2_.

Purified astrocytes were plated on flasks and the culture medium was changed every 2 days. Before the experiments, astrocytes were trypsinized (Sigma‐Aldrich, St. Louis, MO, USA, Cat. No. T4799) and transferred onto cover glasses coated with poly‐L‐lysine (Sigma‐Aldrich, St. Louis, MO, USA, Cat. No. P‐1524). For electrophysiologic experiments, cells were seeded at a lower density to minimize intracellular contacts. Seeded cells were maintained in a feeding medium until experimentation.

### 2.2 Cannabinoid Receptors Labeling

**FIGURE 2:**
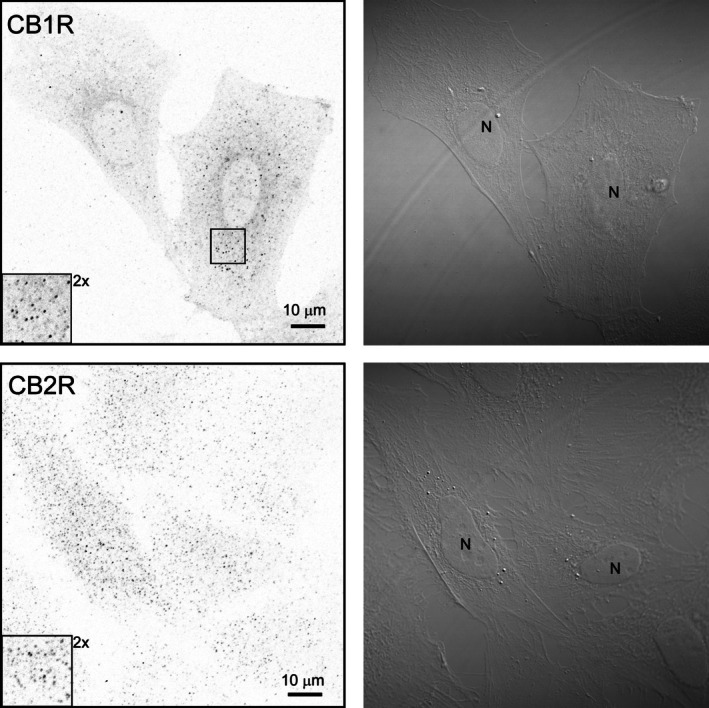
Immunocytochemical detection of CB1R and CB2R. The left panels display confocal microscopy images of astrocytes, fluorescently labeled with antibodies against CB1R (top) and CB2R (bottom). In these grayscale renderings, higher fluorescence intensity appears dark/black, whereas lower signal and background appear light/white. A 2× magnified view of the marked region is shown in the bottom‐left inset. The corresponding transmitted light images are shown in the right panels. N, nucleus.

Confirmation of the presence of cannabinoid receptors on astrocytic membranes was achieved through immunolabeling, resulting in fluorescent signals over the cellular plasmalemma indicative of CB1R expression, however the CB2R was low or ambiguous (Figure 2). Cultured astrocytes were subjected to immunolabeling using rabbit polyclonal anti‐cannabinoid receptor 1 antibody (Abcam, Cambridge, UK, Cat. No. ab23703) at a dilution of 1:200 and rabbit polyclonal anti‐cannabinoid receptor 2 antibody (Abcam, Cambridge, UK, Cat. No. ab3561) at a dilution of 1:500. The astrocytes were then washed in phosphate‐buffered saline (PBS; Invitrogen, Carlsbad, CA, USA, Cat. No. 10010015), fixed, and permeabilized in a 4% formaldehyde (Sigma‐Aldrich, St. Louis, MO, USA, Cat. No. 252549) solution for 15 min at room temperature (22°C–25°C). Subsequently, they were incubated in a blocking buffer (3% bovine serum albumin (Sigma‐Aldrich, St. Louis, MO, USA, Cat. No. A‐6003) and 10% goat serum (Sigma‐Aldrich, St. Louis, MO, USA, Cat. No. G‐6767) in PBS) for 1 h at 37°C to prevent background staining, followed by labeling overnight with primary antibodies against CB1R or CB2R at 4°C. After washing with PBS, secondary antibodies goat anti‐rabbit immunoglobulin G conjugated to fluorescent dye Alexa Fluor 488 (Invitrogen, Carlsbad, CA, USA, Cat. No. A‐11008) were applied at a dilution of 1:600 for 45 min at 37°C. Samples were then mounted onto glass slides using Slowfade Gold antifade reagent (Invitrogen, Carlsbad, CA, USA, Cat. No. S36940). Control experiments using secondary antibodies only, without primary antibodies, were performed in parallel and yielded no detectable signal, confirming the specificity of the immunolabeling.

### 2.3 FRET Measurements

The Förster resonance energy transfer (FRET) microscopy technique relies on energy transfer between fluorescent proteins of nanosensors within transfected cells, where changes in the ratio are monitored using a fluorescent microscope. This method was used to track live intracellular concentrations of L‐lactate ([L‐lactate]_i_ (Sigma‐Aldrich, St. Louis, MO, USA, Cat. No. 71718)) and D‐glucose and ([D‐glucose]_i_ (Sigma‐Aldrich, St. Louis, MO, USA, Cat. No. G‐8270)) in single astrocytes.

The extracellular solutions contained 135.3 mM NaCl (Sigma‐Aldrich, St. Louis, MO, USA, Cat. No. S‐7653), 5 mM KCl (Kemika, Zagreb, Croatia, Cat. No. 1120907), 10 mM HEPES (4‐(2‐hydroxyethyl)‐1‐piperazineethanesulfonic acid) (Sigma‐Aldrich, St. Louis, MO, USA, Cat. No. H‐3375), 0.5 mM NaH_2_PO_4_·H_2_O (Sigma‐Aldrich, St. Louis, MO, USA, Cat. No. S0751), 5 mM NaHCO_3_ (Sigma‐Aldrich, St. Louis, MO, USA, Cat. No. S‐5761), 2 mM MgCl_2_ (Sigma‐Aldrich, St. Louis, MO, USA, Cat. No. M‐8266), 1.8 mM CaCl_2_ (Sigma‐Aldrich, St. Louis, MO, USA, Cat. No. 21115), and 3 mM D‐glucose; pH adjusted to 7.2 with NaOH (Merck, Darmstadt, Germany, Cat. No. 1064981000). Osmolarity ranged from 290 to 310 mOsm, measured with a freezing‐point osmometer (Osmomat030; Gonotech GmbH, Berlin, Germany). Cells were transfected with a genetically encoded FRET‐based lactate nanosensor Laconic (Addgene, Watertown, MA, USA; Plasmid Cat. No. 44238; RRID:Addgene_44 238) at least 16 h before the experiments or with a glucose nanosensor FLII^12^Pglu‐700μδ6 (Addgene, Watertown, MA, USA; Plasmid Cat. No. 17866, RRID:Addgene_17 866). Successful transfection was achieved using FuGENE 6 Transfection Reagent (Promega, Madison, WI, USA, Cat. No. E‐2692) in a lipofection medium devoid of antibiotics or serum.

Changes in the nanosensor fluorescence ratio (ΔFRET) of mTFP/Venus (Laconic) or Citrine‐eYFP/eCFP (FLII^12^Pglu‐700μδ6) were measured in single stimulated astrocytes using a fluorescent microscope (Zeiss Axio Observer.A1; Zeiss, Oberkochen, Germany), equipped with a C‐Apochromat 63×/1.2 NA water objective (Zeiss) and CCD camera (ANDOR Luca S; Andor Technology, Belfast, UK). Excitation was achieved with a Colibri LED module (Zeiss) at 445 nm to elicit fluorescence of the nanosensor, which was then split with an image splitter (Photometrics DV2, Optical Insights, Tucson, AZ, USA) through cyan (460 nm) and yellow (520 nm) filters. Images were acquired every 10 s with a 100‐ms exposure time using Zeiss Zen software. Data analysis was performed using MS Excel and custom code in MATLAB. Figures were compiled for display using Adobe Photoshop, Systat Sigma Plot, and CorelDRAW software. The graphical abstract was created in BioRender by MK (https://BioRender.com/o63c278).

The functionality of successfully transfected astrocytes expressing Laconic or FLII^12^Pglu‐700μδ6 was evaluated during the response to cannabinoid receptor agonists after an initial 300 s baseline with the FRET technique. Before experimentation, cells were incubated for 30 min in the chosen extracellular solution. 200 μL of agonists, including 1 μM Gp1a (Abcam, Cambridge, UK, Cat. No. ab120344) and AM1241 (Sigma‐Aldrich, St. Louis, MO, USA, Cat. No. A6478) (CB2R‐biased ligands previously used as CB2R agonists) for CB2R or 1 μM ACEA (Sigma‐Aldrich, St. Louis, MO, USA, Cat. No. A9719) for CB1R was added at 300 s of measurement. At 900 s, cell viability and sensor functionality were verified with the addition of 10 mM L ‐lactate or 10 mM D‐glucose, depending on the type of transfected sensor.

### 2.4 Electrophysiologic Recordings of Membrane Capacitance

Uncompensated patch‐clamp recordings in whole‐cell configuration were used. Before the experiments, astrocytes cultured on coverslips were maintained in a feeding medium. During recordings, cells were bathed in an extracellular solution containing: NaCl, 131.8 mM; KCl, 5 mM; CaCl_2_, 2.5 mM; MgCl_2_, 2.5 mM; HEPES/NaOH, 10 mM; D‐glucose, 12.5 mM; NaH_2_PO_4_·H_2_O, 0.5 mM; NaHCO_3_, 5 mM; adjusted to pH 7.4 with NaOH. The osmolarity of the solution was 300 ± 10 mOsm determined with a freezing‐point osmometer (Osmomat 3000; Gonotec, Berlin, Germany). Patch‐clamp pipettes, pulled from borosilicate glass capillaries, had a resistance of 2–6 MΩ when filled with electrode solution containing: 140 mM K gluconate (Sigma‐Aldrich, St. Louis, MO, USA, Cat. No. G‐4500), 10 mM tetraethylammonium chloride (Sigma‐Aldrich, St. Louis, MO, USA, Cat. No. T‐2265), 2 mM MgCl_2_, 10 mM HEPES, and 2 mM Na_2_ATP (Sigma‐Aldrich, St. Louis, MO, USA, Cat. No. A‐2383), adjusted to pH 7.2 with KOH (Kemika, Zagreb, Croatia, Cat. No. 11567) and osmolarity 300 ± 10 mOsm. The intracellular calcium concentration [Ca^2+^]_i_ was set to 100 nM using Ca_2_EGTA/EGTA (Sigma‐Aldrich, St. Louis, MO, USA, Cat. No. E‐4378) buffer. All recordings were conducted at room temperature. A volume of 100 μL of the experimental solution was added 100 s after establishing the recording. Control experiments were done by adding the vehicle (extracellular solution). For positive control experiments, 1 mM ATP was used. For experimental solutions, 1 μM ACEA, 1 μM Gp1a, or 1 μM A M1241 was added.

Uncompensated membrane capacitance (*C*_m_) measurements were performed using the SWAM IIC amplifier (Celica, Ljubljana, Slovenia), as previously described (Rituper et al. 2013). The amplifier operated at an 800 Hz lock‐in frequency (sine wave of 1.1 mV rms). Cells were voltage‐clamped at a holding potential of −70 mV. The direct current (*I*_DC_), holding potential (*V*), real (*Y*_Re_), and imaginary (*Y*_Im_) components of the admittance signals were acquired every 50 ms, digitized, and stored on a computer using CELL software (Celica, Ljubljana, Slovenia). The membrane capacitance (*C*_m_), parallel combination between membrane conductance and membrane leak (*G*_m_), and access conductance (*G*_a_) were calculated. Recordings were analyzed using custom‐made software CellAn (Celica, Ljubljana, Slovenia) for MATLAB. Recordings with an access conductance of less than 50 nS were not included in the analysis.

### 2.5 Cytosolic Ca^2+^ Measurements

To record Ca^2+^ fluctuations after cannabinoid stimulation, plated cells were stained with the Ca^2+^‐sensitive fluorescent dye Cal520‐AM (Abcam, Cambridge, UK, Cat. No. ab171868). This dye was added at a concentration of 5 μM in feeding medium and the cells were incubated at 37°C for 20–30 min. After incubation, the medium was replaced with the extracellular solution, and the cells were incubated for another 30 min before recording. Using a fluorescent microscope (Axio Observer.A1) equipped with a C‐Apochromat 63×/1.2 NA objective, the dye was excited with a Colibri LED module emitting 475 nm excitation light. Images were acquired every 3 s with an 80‐ms exposure time and 3% LED intensity for 20 min. The cells were stimulated after 5 min with the addition of 200 μL of 1 μM Gp1a, 1 μM A M1241, or 1 μM ACEA. After 15 min, cell viability was assessed by adding 10 μM ionomycin (Sigma‐Aldrich, St. Louis, MO, USA, Cat. No. I‐0634). To measure calcium spike delays, the time point at which fluorescence intensity reached 50% of the average maximum increase was identified.

### 2.6 Periodic Acid‐Schiff Staining

For the quantification of glycogen in the cytosol, a periodic acid‐Schiff (PAS) kit (Sigma‐Aldrich, St. Louis, MO, USA, Cat. No. 395B‐1KT) with the modified PAS method was used, as described previously (Fink et al. 2021). Cells were plated onto 22‐mm‐diameter poly‐L‐lysine‐coated coverslips 2 days before the experiment. Following treatments (30 min preincubation in 3 mM D‐glucose (control), 1 μM ACEA, or 1 μM A M1241), cells were washed with ice‐cold PBS and fixed for 15 min with 4% paraformaldehyde (Sigma‐Aldrich, St. Louis, MO, USA, Cat. No. P‐6148) in PBS and 10 min with 4% paraformaldehyde with 0.1% Triton X‐100 (Sigma‐Aldrich, St. Louis, MO, USA, Cat. No. T‐9284) at room temperature and washed four times with PBS. For negative control, untreated, fixed and permeabilized astrocytes were exposed to 100 μg/mL diastase (Sigma‐Aldrich, St. Louis, MO, USA, Cat. No. 1.03604) for 10 min at 37°C and stained in parallel with treated coverslips as follows. Cells were incubated for 5 min at room temperature in 1% (w/v) periodic acid solution, then washed under running deionized water and stained for 30 min at room temperature in Schiff's reagent. After staining, the cells were rinsed under running deionized water, dehydrated in alcohol series from 70% to absolute alcohol, and mounted with Eukitt resin (Sigma‐Aldrich, St. Louis, MO, USA, Cat. No. 3989).

Images were acquired using the same setup as described for the FRET measurements, using a Plan‐Apochromat 20×/0.8 NA air objective (Zeiss) and a CCD camera (ANDOR Luca S). Cells were excited using a Colibri LED module at 505 nm with 20% light intensity. The light was collected through the 570–640 nm emission filter. The entire coverslip was systematically sampled, and cells were analyzed on the sampled images, excluding the nucleus. For quantitative analysis, imaging software Zen (Zeiss) and Fiji were used (Schindelin et al. 2012). The mean fluorescence intensity of PAS staining per cell was determined by averaging the fluorescence intensities of all pixels in the region of interest corresponding to the cell cross‐sectional area.

### 2.7 Quantitative Real‐Time PCR (RT‐qPCR)

Total RNA was extracted from cultured rat astrocytes using the PureLink RNA Mini Kit (Thermo Fisher Scientific/Invitrogen, Cat. 12183018A) following the manufacturer's protocol, including on‐column genomic DNA removal with the PureLink DNase Set (Cat. 12185010). Samples were homogenized using the PureLink Homogenizer, 50 Rxn (Cat. 12183026). RNA concentration and purity were determined by micro‐volume absorbance (A260/A280) on an Epoch spectrophotometer (BioTek Instruments Inc., USA). Total RNA was reverse‐transcribed in 20 μL using the High‐Capacity cDNA Reverse Transcription Kit (Applied Biosystems/Thermo Fisher Scientific, Cat. 4368814) with RNase Inhibitor (Cat. N8080119) on a PTC‐100 Programmable Thermal Controller (MJ Research). No‐reverse‐transcriptase (−RT) controls were prepared.

Reactions were run on a QuantStudio 3 Real‐Time PCR System (Applied Biosystems) using TaqMan Universal Master Mix II, with UNG (Thermo Fisher Scientific, Cat. 4440038) and TaqMan Gene Expression Assays for rat Cnr1 (Rn00562880_m1) and Cnr2 (Rn01637601_m1). Ppia/cyclophilin A (Rn00690933_m1) and Rplp0 (Rn03302271_gH) served as reference genes. Reactions (10 μL final) contained 1× Master Mix, 1× assay, and 1–2 μL cDNA (typically a 1:10 dilution of RT product). Plates were Applied Biosystems 96‐Well Fast Thermal Cycling Plates (Cat. 4346907) sealed with MicroAmp Optical Adhesive Film (Cat. 4311971). Each biological sample was run in technical duplicate; no‐template controls (NTC) and ‐RT controls were included on every plate.

Technical replicates with Ct spread > 0.5 were inspected and outliers removed if attributable to sealing/pipetting artifacts. Gene expression was normalized to the geometric mean of Ppia and Rplp0. Relative expression was calculated as (1 + E_reference_)^Ct,reference^/(1 + E_target gene_)^Ct,target gene^ where E is the PCR efficiency and Ct is the threshold cycle estimated with LinRegPCR (2018.0) from raw fluorescence data. Nondetection was defined a priori as no amplification or Ct ≥ 40 in all technical replicates together with negative NTC and ‐RT controls.

### 2.8 Statistical Analysis

The results are presented as means ± standard error of the mean unless otherwise specified. Normality of each data set was assessed with the Shapiro–Wilk test in Sigma Plot 11.0. All data sets fail the normality criterion (*p* < 0.05) and were analyzed using Kruskal–Wallis one‐way analysis of variance (ANOVA) on ranks (Kruskal–Wallis ANOVA), followed by post hoc Dunn's test for all pairwise multiple comparisons of the ranked data. Differences were considered significant at *p* < 0.05 and denoted as *p < 0.05, **p < 0.01, ***p < 0.001; nonsignificant differences are indicated as n.s. (*p* > 0.05). Statistical analyses were conducted using Sigma Plot 11.0, MATLAB R2001b, and MS Excel 2010 software. Graphical representations were constructed as box plots, with the box representing the middle 50% of the data, the mean depicted by the red line, and the median by the black line within the box. Whiskers extending above and below the box indicate the 90th and 10th percentiles, respectively. Sample sizes are reported adjacent to the boxes. No predetermined statistical method was used to establish the sample size, and neither blinding nor randomization procedures were used. In addition, no outlier tests were conducted. Graphs were generated using Sigma Plot 11.0 and Adobe Photoshop software.

## 3 Results

### 3.1 CB1R Are Present on the Astrocytic Membrane

Although CB1R expression on astrocytic membranes is well established (Navarrete et al. 2014; Han et al. 2012), we assessed CB1R and CB2R in our cultured astrocytes using immunolabeling and RT‐qPCR.

We used assays specific for rat Cnr1 (Rn00562880_m1) and Cnr2 (Rn01637601_m1). Expression values were normalized to the geometric mean of two endogenous controls from our reagent set, Ppia (cyclophilin A; Rn00690933_m1) and Rplp0 (large ribosomal protein P0; Rn03302271_gH). Across independent RNA preparations, Cnr1 was readily detected in all samples, whereas Cnr2 was not detected (Ct undetermined at 40 cycles), matching the behavior of negative controls. Assay performance was validated in a rat blood sample, which showed a clear Cnr2 signal (Ct: 36), confirming that the assay could detect low Cnr2 expression under our conditions. No‐template and no‐reverse‐transcriptase controls were uniformly negative, and Ppia/Rplp0 showed stable amplification across samples. These data indicate that, under our culture conditions, astrocytes express CB1 transcripts but lack detectable CB2 transcripts. Accordingly, in subsequent sections we interpret pharmacological responses to ligands commonly used as CB2 agonists with caution and refer to them as ligand‐evoked (likely CB1‐linked or off‐target under our conditions) rather than CB2‐mediated.

Laser‐scanning confocal microscopy revealed clear, punctate CB1R labeling on astrocytic membranes (Figure 2). A faint, punctate signal was observed with the CB2R antibody; however, RT‐qPCR detected Cnr1 transcripts but failed to detect Cnr2 under our culture conditions. Control experiments without primary antibodies showed no detectable fluorescence (data not shown). Given the absence of Cnr2 transcripts, we treat the CB2R‐like immunosignal as ambiguous and refrain from inferring CB2R expression. Thus, our data support CB1R presence in these preparations, whereas CB2R appears unlikely, in line with the ongoing debate regarding astrocytic CB2R (Kofalvi et al. 2016).

### 3.2 Cannabinoid Receptor Stimulation Increases Astrocytic [L‐Lactate]_i_ and [D‐Glucose]_i_

**FIGURE 3:**
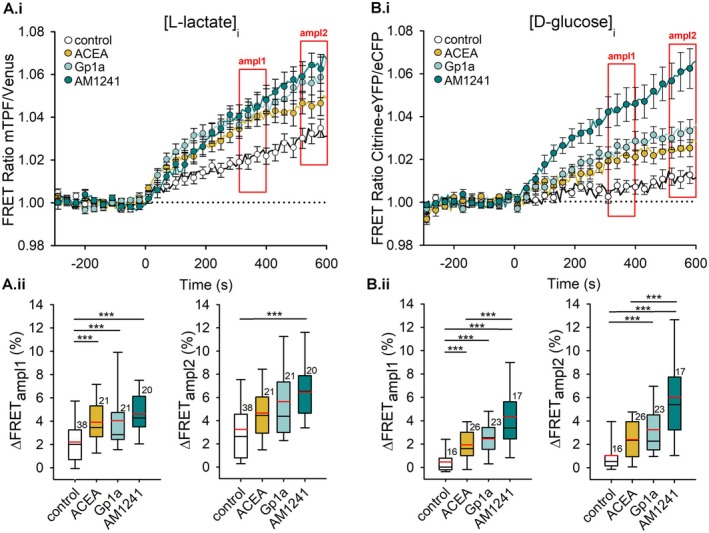
Time‐dependent increase in intracellular L‐lactate ([L‐lactate]_i_) and D‐glucose ([D‐glucose]_i_) on cannabinoid receptor activation. Astrocytes were transfected with either the Laconic nanosensor (A, left panel) to monitor [L‐lactate]_i_ or the FLII12Pglu‐700μδ6 nanosensor (B, right panel) to measure [D‐glucose]_i_. Cells were stimulated with a control extracellular solution, 1 μM ACEA (CB1R agonist), and 1 μM CB2‐biased ligands Gp1a or AM1241. (Ai) Changes in [L‐lactate]_i_ were quantified at two time points: 400 s (ampl1) and 600 s (ampl2) (marked by red rectangles). (Aii) Stimulation with cannabinoid receptor agonists significantly increased [L‐lactate]_i_ at ampl1 (***p ≤ 0.001; left box plot). At ampl2, the increase remained significant only after AM1241 stimulation (***p ≤ 0.001; right box plot). (Bi) Change in [D‐glucose]_i_ was measured at the same time points: 400 s (ampl1) and 600 s (ampl2) (red rectangles). (Bii) Stimulation with all ligands resulted in a significant increase in [D‐glucose]_i_ at ampl1 (***p ≤ 0.001; left box plot). At ampl2, only Gp1a and AM1241 ligands maintained a significant effect on [D‐glucose]_i_ levels (***p ≤ 0.001; right box plot). The number of independent experiments (coverslips) is indicated on the boxes. Data were analyzed using Kruskal–Wallis ANOVA, followed by Dunn's post hoc test.

To assess astrocytic metabolite dynamics under different stimulations, intracellular [L‐lactate]_i_ and [D‐glucose]_i_ concentrations were measured using nanosensor‐based fluorescence changes and FRET calculations. Specifically, the FLII^12^Pglu‐700μδ6 nanosensor for glucose and Laconic for L‐lactate were utilized. Astrocytes were stimulated with 1 μM ACEA (a CB1R agonist) or 1 μM CB2‐biased ligands Gp1a and AM1241. Each cell underwent stimulation after a 5‐min baseline recording (time 0 in Figure 3, Ai and Bi) and was monitored for 900 s after stimulation. In the final 300 s, cell viability and nanosensor functionality were validated with a 10 mM bolus of L‐lactate or D‐glucose, depending on the nanosensor used (data not shown).

Measurements were performed using a Zeiss Colibri fluorescence microscope with LED illumination. Due to the system's high sensitivity and the mechanical responsiveness of the cells (Turovsky et al. 2020), control experiments revealed a small increase in the FRET ratio of nanosensors for both L‐lactate and D‐glucose on the addition of extracellular solution (control). The FRET ratio increased by 3.24% ± 0.44% for L‐lactate (*n* = 38) and 1.04% ± 0.55% for D‐glucose (*n* = 16).

Activation of CB1R by ACEA led to a significant increase in the FRET ratio at ampl1 for both metabolites. Specifically, the FRET ratio increased by 3.91% ± 0.45% for [L‐lactate]_i_ (*n* = 21, Dunn's post hoc test, *p* < 0.05, *Q* = 2.95) and by 1.93% ± 0.31% for [D‐glucose]_i_ (*n* = 26, Dunn's post hoc test, *p* < 0.05, *Q* = 2.67). However, at ampl2, the effects were not statistically significant compared with the control; the FRET ratio increased by 4.67% ± 0.54% for [L‐lactate]_i_ and by 2.40% ± 0.34% for [D‐glucose]_i_ (Kruskal–Wallis ANOVA, Dunn's post hoc test, *p* > 0.05, *Q* = 1.97, *Q* = 2.34, respectively).

Application of the CB2‐biased ligands Gp1a and AM1241 produced similar effects. At ampl1, AM1241 significantly increased the FRET ratio for both metabolites: 4.62% ± 0.49% for [L‐lactate]_i_ (*n* = 20, Kruskal–Wallis ANOVA, *p* ≤ 0.001, *H* = 20.73, df = 3, Dunn's post hoc test, *p* < 0.05, *Q* = 4.18) compared with the control increase of 2.22% ± 0.34% (*n* = 38), and 4.35% ± 0.73% for [D‐glucose]_i_ (*n* = 17, Dunn's post hoc test, *p* < 0.05, *Q* = 5.07). Gp1a also induced a significant increase: 4.04% ± 0.61% for [L‐lactate]_i_ (Kruskal–Wallis ANOVA, *p* ≤ 0.001, *H* = 20.73, df = 3, Dunn's post hoc test, *p* < 0.05, *Q* = 2.66) and 2.45% ± 0.34% for [D‐glucose]_i_ (Kruskal–Wallis ANOVA, *p* ≤ 0.001, *H* = 27.00, df = 3, Dunn's post hoc test, *p* < 0.05, *Q* = 3.57). At ampl2, only AM1241 sustained a significant increase in the FRET ratio for both metabolites, increasing to 6.47% ± 0.62% for [L‐lactate]_i_ (*n* = 20, Kruskal–Wallis ANOVA, *p* ≤ 0.001, *H* = 17.50, df = 3, Dunn's post hoc test, *p* < 0.05, *Q* = 4.00) and 6.00% ± 0.88% for glucose (*n* = 17, Kruskal–Wallis ANOVA, *p* ≤ 0.001, *H* = 27.11, df = 3, Dunn's post hoc test, *p* < 0.05, *Q* = 5.10). Gp1a significantly increased [D‐glucose]_i_ levels (3.25% ± 0.51%, Kruskal–Wallis ANOVA, *p* ≤ 0.001, *H* = 27.11, df = 3, *post hoc* Dunn's method, *p* < 0.05, *Q* = 3.18), but the increase in [L‐lactate]_i_ (5.65% ± 0.85%) was not statistically significant (Kruskal–Wallis ANOVA, Dunn's post hoc test, *p* > 0.05, *Q* = 2.52).

These findings underscore the distinct effects of the ACEA, Gp1a and AM1241 ligands on [L‐lactate]_i_ and [D‐glucose]_i_ levels in astrocytes; AM1241 elicited the relatively strongest response.

### 3.3 Glycogen Stores Are Depleted After Cannabinoid Receptor Stimulation

**FIGURE 4:**
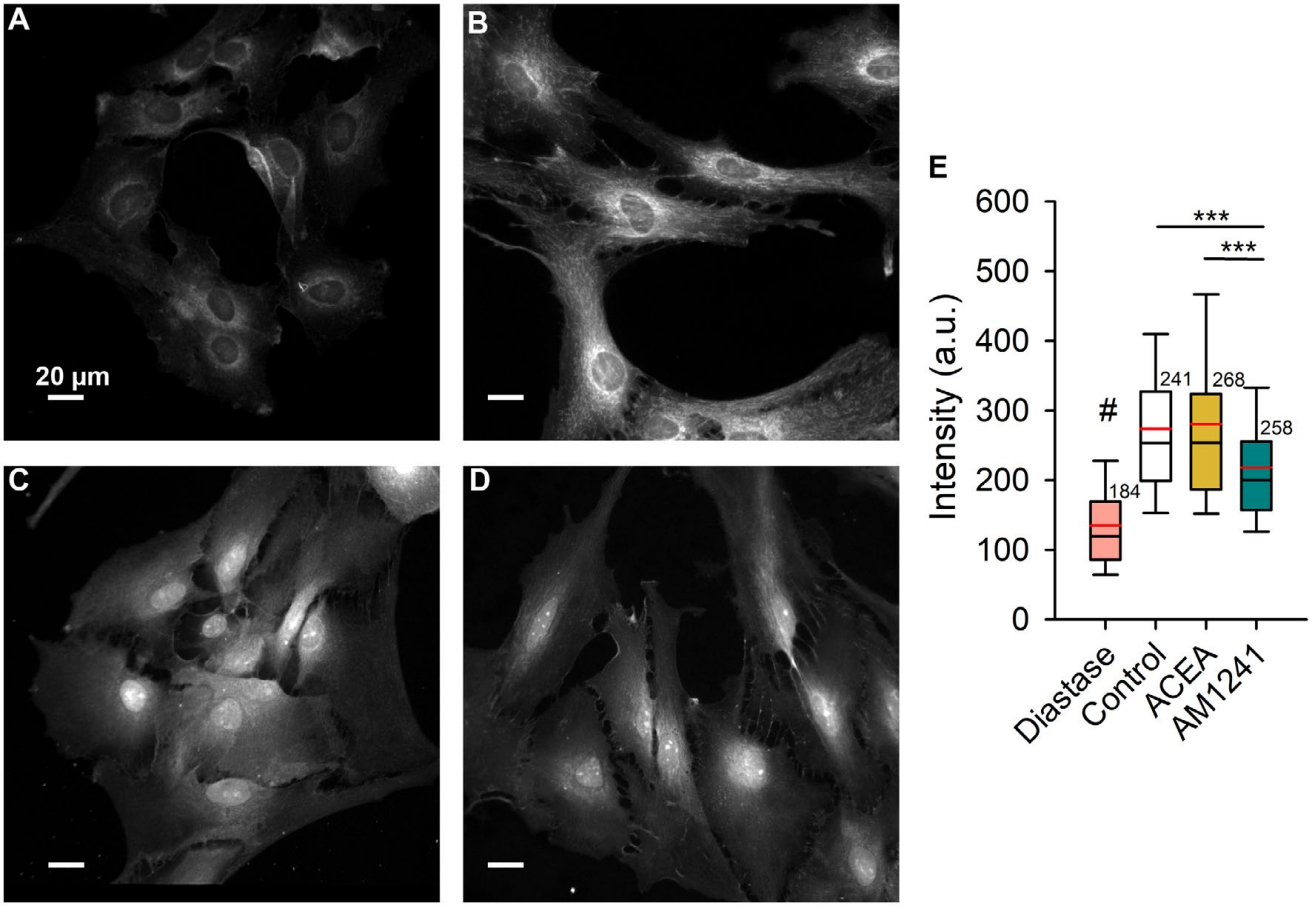
Activation with ACEA (CB1R ligand) and AM1241 (CB2R‐biased ligand) of glycogen labeled astrocytes. Astrocytes stained with periodic acid‐Schiff (PAS) were treated with (A) diastase (fixed and permeabilized cells) or pretreated with (B) control extracellular solution (3 mM glucose), (C) 1 μM ACEA (CB1R agonist), and (D) 1 μM A M1241 (CB2R‐biased ligand). (E) Quantitative analysis revealed that all treatments differed significantly from the diastase condition (#*p* ≤ 0.001). In addition, AM1241‐treated cells exhibited a significant reduction in glycogen content compared with both the control and ACEA‐treated cells (***p ≤ 0.001). Micrographs were acquired using a 20× objective; white scale bars, 20 μm.

To quantify glycogen content, the fluorescence emission light intensity in the cytoplasm, expressed in arbitrary units (a.u.) was analyzed (Figure 4). Astrocytes were pretreated at 37°C with three different solutions for 30 min. The treatment conditions were as follows: 3 mM glucose extracellular solution (with and without 100 μg/mL diastase after fixation and permeabilization) for controls, 1 μM ACEA for CB1R activation, and 1 μM of the CB2R‐biased ligand AM1241. Coverslips were subsequently fixed in formaldehyde and stained using the PAS method (see Section 2.6). Diastase treatment, which degrades glycogen, resulted in a significant reduction in intensity (134.92 ± 5.47 a.u., *n* = 184) compared with the control (274.10 ± 7.27 a.u., *n* = 241; *p* ≤ 0.001, Kruskal–Wallis ANOVA, *H* = 272.50, Dunn's post hoc test, *Q* = 14.67). CB1R stimulation (280.86 ± 8.22 a.u., *n* = 268) did not significantly differ from the control, indicating no significant effect on glycogen stores. However, AM1241 stimulation led to a significant decrease in glycogen content (218.38 ± 5.46 a.u., *n* = 258; *p* < 0.001, Dunn's post hoc test, *Q* = 5.78 versus control and *Q* = 5.41 versus CB1R stimulation).

In astrocytes stimulated with ACEA (CB1R ligand) (Figure 4C) and AM1241 (CB2R‐biased ligand) (Figure 4D), morphologic changes resembling a reactive‐like phenotype with more pronounced processes compared with controls were observed. These observations suggest that cannabinoid receptor activation may influence astrocytic membrane dynamics, which is further explored in the next section.

### 3.4 Cannabinoid Receptor Stimulation Promotes Exocytosis

**FIGURE 5:**
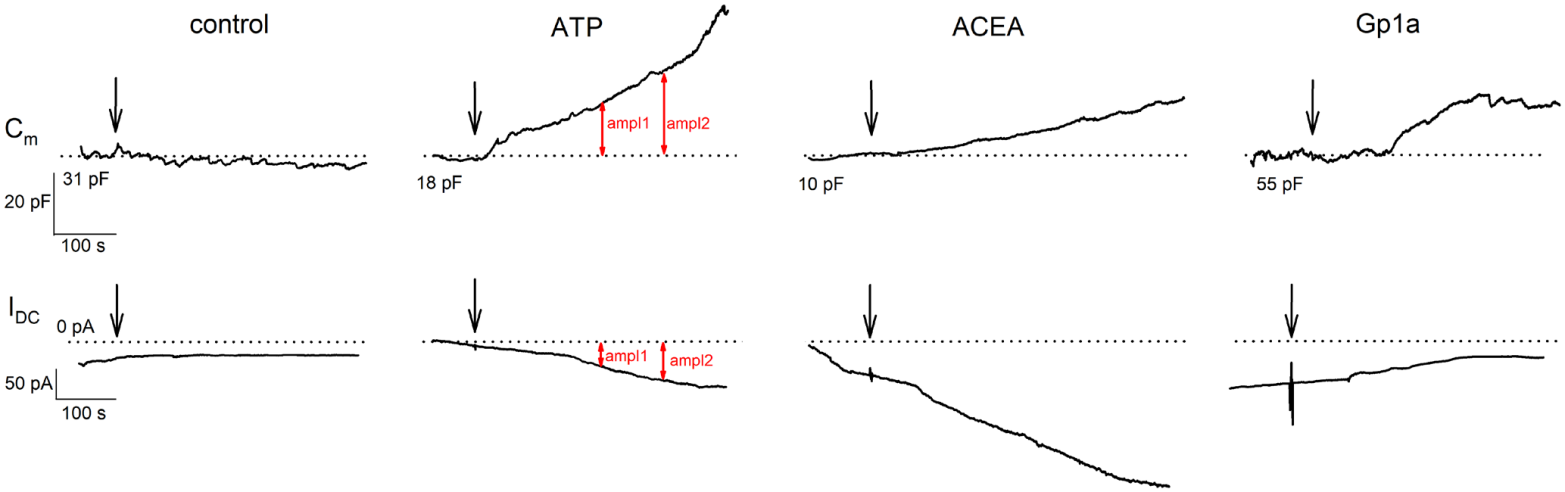
Representative whole‐cell electrophysiologic recordings of astrocyte membrane capacitance. Traces of membrane capacitance (*C*_m_) and current (*I*_DC_) recorded in four experimental conditions. From left to right: Control condition, 1 mM ATP stimulation, 1 μM ACEA stimulation, and 1 μM Gp1a stimulation. The dotted horizontal line denotes the baseline for the *C*_m_ value before stimulation and 0 pA for the current. Red arrows indicate the two points where amplitude measurements were taken.

To investigate whether cannabinoid signaling influences astrocyte plasma membrane dynamics, membrane capacitance (*C*_m_), a parameter related to the area of the plasma membrane, was measured (Neher and Marty 1982), using the whole‐cell patch‐clamp technique after acute stimulation with different solutions. Astrocytes were exposed to the extracellular solution (negative control), ATP (1 mM, positive control), ACEA (1 μM, CB1R agonist), Gp1a or AM1241 (1 μM, CB2R‐biased ligands). Figure 5 shows representative traces of *C*_m_ and direct current (*I*_DC_) across experimental conditions. All parameters were evaluated at two time points: amplitude 1 (150–200 s after stimulation) and amplitude 2 (250–300 s after stimulation).

**FIGURE 6:**
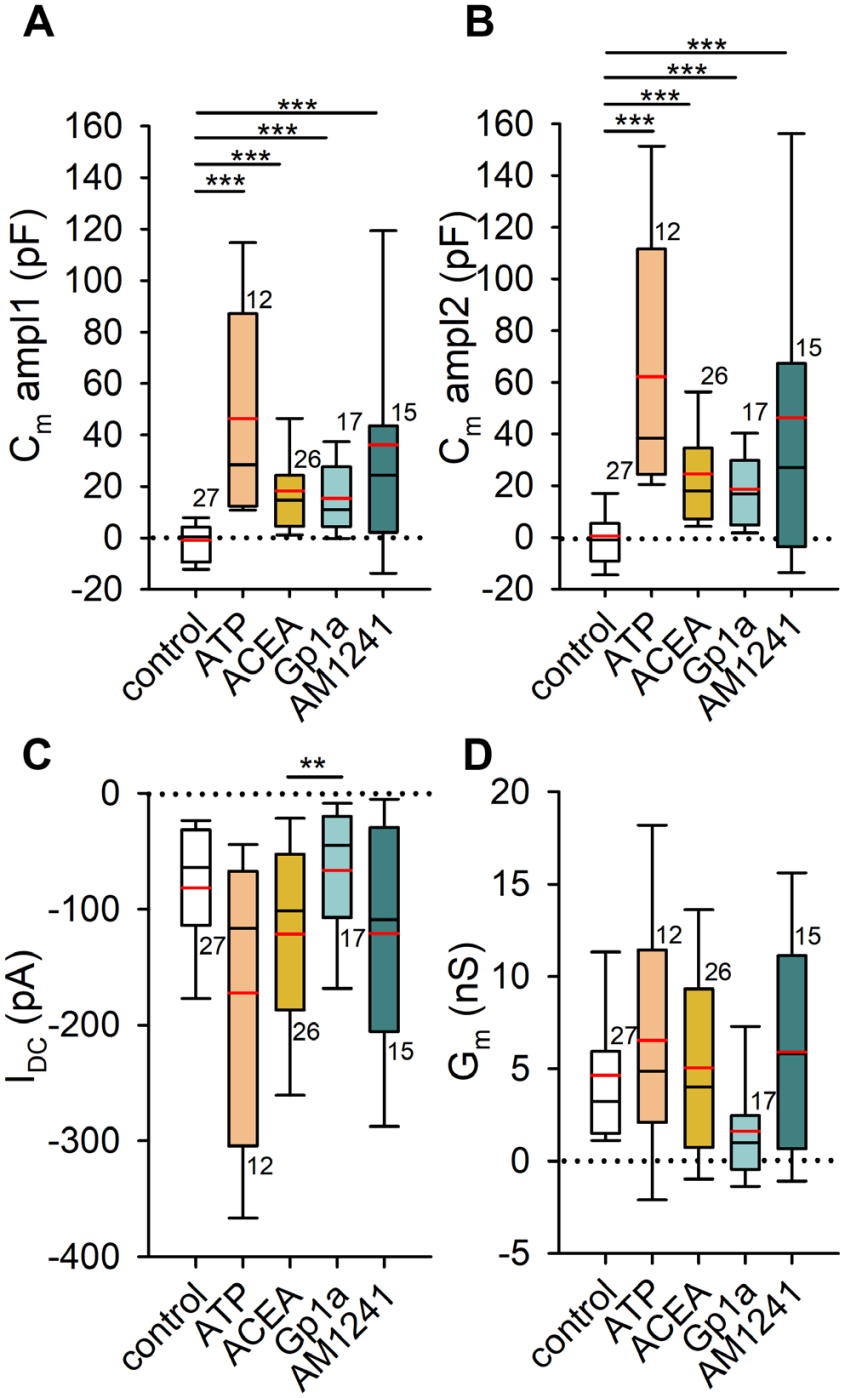
Cannabinoid receptor agonist stimulation increases astrocyte membrane capacitance (*C*_m_). (A, B) *C*_m_ measured in pF after the addition of extracellular solution (negative control), 1 mM ATP (positive control), 1 μM ACEA (CB1R agonist), 1 μM Gp1a and 1 μM A M1241 (CB2R‐biased ligands). Measurements were taken at two time points: 200 s (ampl1, A) and 300 s (ampl2, B). Both ATP and cannabinoid receptor agonists induced a statistically significant increase in *C*_m_ (***p ≤ 0.001). The number of cells (on separate coverslips) analyzed is indicated next to the boxplots. (C) Current (*I*_DC_) across the plasma membrane was not significantly affected by cannabinoid receptor agonists, whereas ATP stimulation led to a significant increase in inward current amplitude (−172.23 ± 35.80 pA) compared with stimulation by 1 μM Gp1a (−66.16 ± 13.68 pA, **p = 0.033; Kruskal–Wallis one‐way ANOVA, *H* = 10.50, df = 4, Dunn's post hoc test, *Q* = 2.87). Black horizontal lines indicate mean current amplitudes, while red horizontal lines denote median values. The number of cells (on separate coverslips) analyzed is shown next to the boxplots. Amplitudes were measured between 250 and 300 s of recordings. (D) Membrane conductance (*G*_m_) was not significantly different between the tested groups.

As a positive control, ATP was used to confirm the expected increase in *C*_m_, consistent with previous findings that extracellular ATP induces exocytosis in astrocytes (Pangrsic et al. 2006). On the addition of ATP, membrane conductance (*G*_m_) increased to 6.55 ± 1.90 nS (Figure 6D, orange bar), and the average current reached −172.23 ± 35.80 pA (Figure 6C, orange bar), supporting its role in promoting membrane dynamics.

Figure 6A,B present membrane capacitance (*C*_m_) values in direct measurements (pF). For comparison, capacitance changes were also normalized. Values were obtained at two time points as previously defined: ampl1 (Figure 6A) and ampl2 (Figure 6B). In control experiments, astrocytic capacitance remained unchanged after the addition of extracellular solution (ampl1: −0.89 ± 1.82 pF or −0.44% ± 2.83%; ampl2: 0.63 ± 2.83 pF or 3.22% ± 2.90%). In contrast, ATP stimulation induced a significant increase in *C*_m_, consistent with enhanced exocytotic activity, reaching 46.29 ± 11.62 pF or 63.71% ± 11.98% in ampl1 (Kruskal–Wallis ANOVA, *p* ≤ 0.001, *H* = 36.20, df = 4, *post hoc* Dunn's method, *p* < 0.05, *Q* = 5.12 for nonnormalized measurements in pF or *H* = 43.22, *Q* = 5.32) for normalized data in %, and 62.23 ± 14.46 pF or 89.22% ± 15.92% in ampl2 (Kruskal–Wallis ANOVA, *p* ≤ 0.001, *H* = 37.55, df = 4, *post hoc* Dunn's method, *p* < 0.05, *Q* = 5.49 or *H* = 42.94, *Q* = 5.26). Stimulation of CB1R with ACEA and application of the CB2‐biased ligands Gp1a and AM1241 each resulted in a statistically significant increase in *C*_m_. Activation of CB1R with ACEA led to an increase of 18.28 ± 3.85 pF or 45.76% ± 11.66% in ampl1 (Kruskal–Wallis ANOVA, *p* ≤ 0.001, *H* = 36.10, df = 4, *post hoc* Dunn's method, *p* < 0.05, *Q* = 4.08 or *H* = 43.22, *Q* = 4.86), and 24.49 ± 4.34 pF or 61.85% ± 12.88% in ampl2 (Kruskal–Wallis ANOVA, *p* ≤ 0.001, *H* = 37.55, df = 4, *post hoc* Dunn's method, *p* < 0.05, *Q* = 4.18 or *H* = 42.94, *Q* = 4.89). Similarly, the CB2‐biased agonist Gp1a increased *C*_m_ by 15.28 ± 3.25 pF or 31.00% ± 8.57% in ampl1 (Kruskal–Wallis ANOVA, *p* ≤ 0.001, *H* = 36.20, df = 4, *post hoc* Dunn's method, *p* < 0.05, *Q* = 3.42 or *H* = 43.22, *Q* = 3.93), and 18.70 ± 3.13 pF or 41.47% ± 10.31% in ampl2 (Kruskal–Wallis ANOVA, *p* ≤ 0.001, *H* = 37.55, df = 4, *post hoc* Dunn's method, *p* < 0.05, *Q* = 3.21 or *H* = 42.94, *Q* = 3.65). Application of AM1241 (1 μM) under the same recording conditions also increased C_m_, reaching 36.14 ± 11.53 pF (12.59% ± 5.46%, *n* = 15) in ampl1 and 36.14 ± 15.34 pF (19.29% ± 9.17%, *n* = 15) in ampl2; in the nonnormalized data, ΔC_m_ values were significantly higher than in control cells at both time points (*p* ≤ 0.05). Taken together, ACEA, Gp1a, and AM1241 all promoted stimulus‐evoked increases in astrocyte C_m_ consistent with enhanced exocytotic activity. Given that Cnr2 transcripts were undetectable in these cultures, we describe the Gp1a‐ and AM1241‐evoked capacitance changes as ligand‐evoked effects at the concentrations used.

Use of CBR agonists did not result in statistically significant changes in *I*_DC_ compared with control conditions (−81.35 ± 10.50 pA, Figure 6C). After CB1R stimulation with ACEA, the mean final current was −121.36 ± 17.19 pA, with a corresponding membrane conductance (*G*_m_) of 5.05 ± 1.07 nS. After stimulation with Gp1a, the final current was −66.16 ± 13.68 pA, with a mean *G*_m_ of 1.62 ± 0.68 nS (Figure 6C and Figure 6D). Application of AM1241 (1 μM) likewise did not produce statistically significant changes in I_DC_ (−120.97 ± 25.87 pA) or Gm (5.90 ± 1.56 nS) compared with control (Figure 6C,D). In contrast, positive control stimulation with 1 mM ATP induced a statistically significant increase in current (−172.24 ± 35.80 pA, Kruskal–Wallis ANOVA, *p* = 0.033, *H* = 10.50, df = 4, *post hoc* Dunn's method, *p* < 0.05, *Q* = 2.87), but not in *G*_m_ compared with stimulation by Gp1a. *G*_m_ was not significantly different in treatments and control experiments.

### 3.5 Intracellular Ca^2+^ Is Increased After Cannabinoid Receptor Activation

**FIGURE 7:**
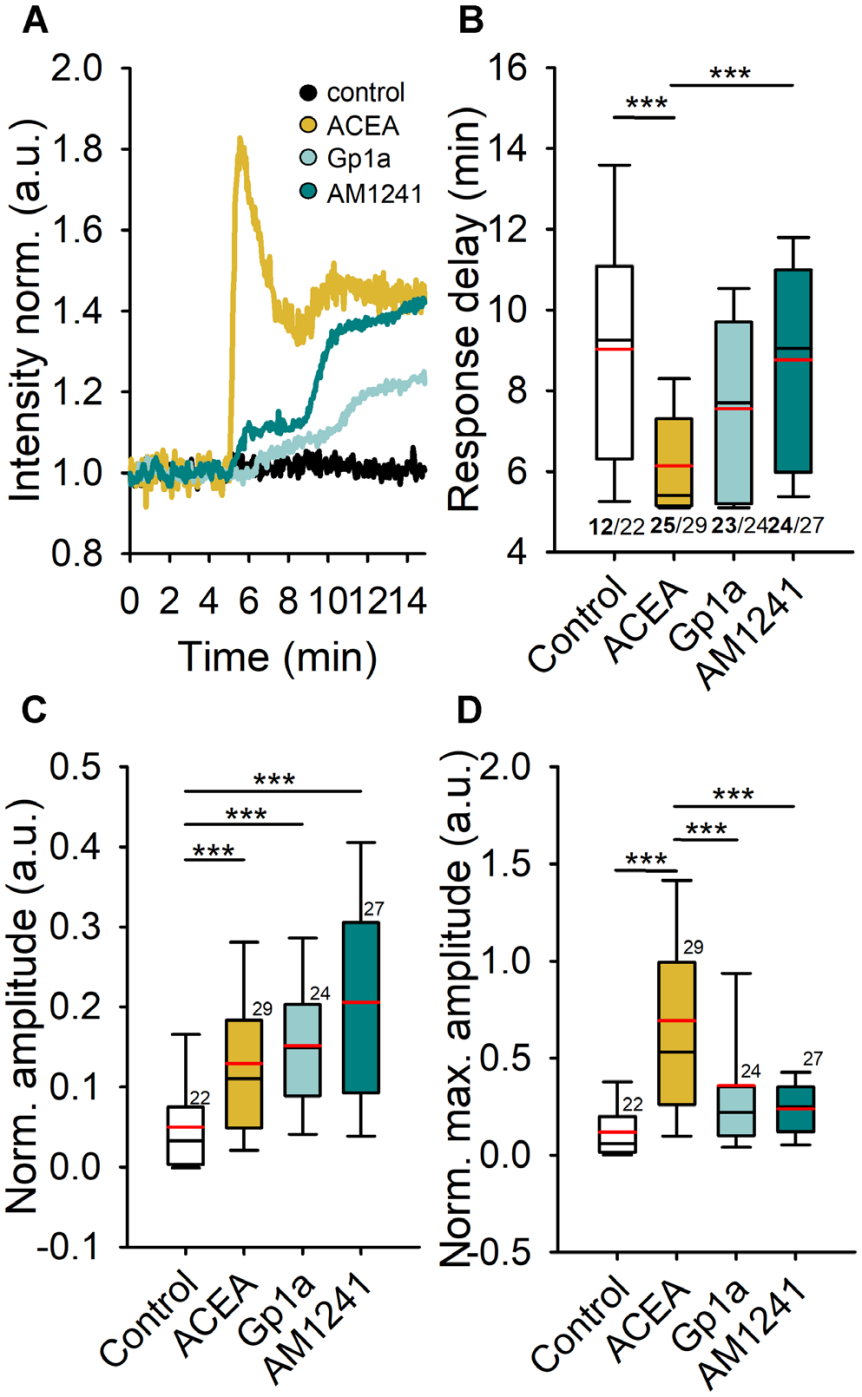
CB1R activation induces a shorter Ca^2+^ response delay and a time‐dependent increase in ([Ca^2+^]_i_). Astrocytes labeled with Cal520‐AM were stimulated with control extracellular solution, 1 μM ACEA (CB1R agonist), 1 μM Gp1a, and 1 μM A M1241 (CB2R biased ligand). (A) Representative traces of Ca^2+^ responses for each treatment, displayed as normalized intensity in arbitrary units (a.u.). The arrow indicates the time of stimulation. (B) Cal520‐AM‐labeled astrocytes exhibited distinct temporal Ca^2+^ dynamics upon ligand addition: ACEA (yellow line and box) resulted in a faster and more pronounced peak Ca^2+^ response (***p ≤ 0.001, *H* = 19.14, df = 3, *Q* = 3.31). The response delay (in minutes) stimulation was significantly shorter compared with the control stimulation with AM1241 (***p ≤ 0.001, *H* = 19.14, df = 3, *Q*_(ACEA/AM1241)_ = 3.90). Only responsive cells were included in delay calculations; the number of responsive cells is shown in bold beneath the bars, indicating the fraction of responsive cells of the total measured. Ca^2+^ amplitudes were measured at two time points: Final amplitude (C) and maximum amplitude (D), both expressed in arbitrary units. In normalized data, all three agonists significantly increased [Ca^2+^]_i_ levels compared with the control (***p ≤ 0.001, *H*_(FINAL)_ = 25.29, *H*_(MAX)_ = 31.13, df = 3).

The increases in Ca^2+^ displayed distinct temporal dynamics after stimulation with 1 μM ACEA (CB1R agonist), 1 μM Gp1a (CB2R ligand), 1 μM A M1241 (CB2R ligand), or extracellular solution (control). To quantify these responses, changes in amplitude from the baseline signal were measured, averaging the final 10 frames recorded 10 min after stimulation. In addition, the maximum amplitude and time to reach maximal change for each recorded cell were determined. Representative traces for each treatment are presented as normalized fluorescence intensity (Figure 7A). Statistical analyses were performed using Kruskal–Wallis ANOVA, followed by Dunn's post hoc test.

The time to reach maximal amplitude differed across treatments when only responsive cells were included in the analysis. In control experiments, 12 of 22 astrocytes responded, presumably due to mechanical stimuli from the addition of a bolus, with a mean response delay of 9.03 ± 0.81 min (Figure 7B, white box). To provide clarity, and because nearly half of the control cells were unresponsive, one representative unresponsive control experiment is shown in Figure 7A (black line). CB1R stimulation with ACEA resulted in a significantly shorter response delay (6.14 ± 0.25 min, 25 of 29 responsive cells), occurring 1.41 min faster than the response to Gp1a (7.55 ± 0.49 min, n.s.) and 2.62 min faster than the response to AM1241 (8.76 ± 0.49 min, 24 of 27 responsive cells, *p* ≤ 0.001, Kruskal–Wallis ANOVA, *H* = 19.14, df = 3), as shown in Figure 7B.

Both CB2R‐biased ligands, Gp1a and AM1241, significantly enhanced [Ca^2+^]_i_ at the end of the recording, with increases of 1.67 ± 0.27 a.u. and 2.05 ± 0.25 a.u., respectively, compared with the control (0.64 ± 0.20 a.u., *p* ≤ 0.001; Kruskal–Wallis ANOVA, *H* = 25.90, df = 3, *post hoc* Dunn's method, *p* < 0.05, *Q*_(Gp1a)_ = 3.63, *Q*_(AM1241)_ = 4.70). In contrast, CB1R activation with ACEA resulted in a [Ca^2+^]_i_ increase of 0.99 ± 0.15 a.u., which was not statistically significant. After normalizing the data, all three cannabinoid receptor agonists significantly increased [Ca^2+^]_i_ levels (Figure 7C), with increases of 12.93% ± 1.96% for ACEA (CB1R agonist, *p* < 0.05, *Q* = 2.94), 15.15% ± 1.78% for Gp1a (CB2R‐biased ligand, *p* < 0.05, *Q* = 3.78), and 20.61% ± 2.57% for AM1241 (CB2R‐biased ligand, *p* < 0.05, *Q* = 4.82) compared with the control (5.01% ± 1.24%, *p* ≤ 0.001, Kruskal–Wallis ANOVA, *H* = 25.29, df = 3, *post hoc* Dunn's method).

Analysis of the maximal amplitude change (Figure 7D) revealed a greater increase in Ca^2+^‐dependent fluorescence after ACEA stimulation than after Gp1a and AM1241 stimulation. ACEA treatment led to an increase in ([Ca^2+^]_i_) by 5.41 ± 1.06 a.u. (*p* < 0.05, *Q* = 4.93), whereas Gp1a and AM1241 increased [Ca^2+^]_i_ by 3.47 ± 0.85 a.u. (*p* < 0.05, *Q* = 2.80) and 2.40 ± 0.27 a.u. (*p* < 0.05, *Q* = 2.66), respectively (all versus control: 1.99 ± 0.85 a.u., *p* ≤ 0.001, Kruskal–Wallis ANOVA, *H* = 24.34, df = 3, *post hoc* Dunn's method). Normalized data confirmed a statistically significant increase in maximal [Ca^2+^]_i_ amplitude after CB1R activation; ACEA treatment resulted in a 69.22% ± 10.35% (*p* < 0.05, *Q* = 5.53) increase compared with 35.82% ± 10.08% after Gp1a and 23.91% ± 2.69% after AM1241 stimulation (control: 11.86% ± 2.84%). The ACEA‐induced increase in maximal amplitude was significantly higher than all other groups (*p* ≤ 0.001, Kruskal–Wallis ANOVA, *H* = 31.13, df = 3).

Our observations raise the possibility that cannabinoid‐induced Ca^2+^ elevations could serve as a common signal linking vesicular gliotransmitter release with metabolic changes in astrocytes.

## 4 Discussion

Cannabinoid receptors, initially identified in GABAergic interneurons for retrograde signaling, are now recognized as some of the most abundant G‐protein‐coupled receptors (GPCRs) in the brain, extending their signaling beyond synapses to intracellular organelles such as mitochondria (Jimenez‐Blasco et al. 2020), where mitochondrial CB1R modulate cellular respiration (Bénard et al. 2012). They also negatively regulate glycolysis and L‐lactate production in astrocytes through multiple mechanisms: reducing astrocytic O_2_ consumption via soluble adenylyl cyclase inhibition, destabilizing mitochondrial complex I, lowering mitochondrial reactive oxygen species levels, and subsequently diminishing hypoxia‐inducible factor 1 signaling, all of which suppress glycolysis and L‐lactate production (Keimpema et al. 2020). Beyond mitochondrial CB1R signaling, activation of CB2R in astrocytes in other models has been reported to reprogram glucose metabolism. Stimulation of CB2R engages AMP‐activated protein kinase, a key energy sensor that responds to elevated AMP/ATP ratios and ([Ca^2+^]_i_) levels (Choi et al. 2013). In astrocytes, AMP‐activated protein kinase activation leads to enhanced glucose uptake and increased alanine production, indicating a shift toward glycolysis (Voss et al. 2020), aligning with evidence that CB1R activation reduces mitochondrial oxidative metabolism (Duarte et al. 2012; Bénard et al. 2012).

Our new RT‐qPCR analyses detected Cnr1 but not Cnr2 in these astrocyte cultures. Consequently, we do not attribute effects of AM1241/Gp1a to CB2R. At 1 μM, these ligands can plausibly engage CB1R or other targets; we therefore interpret their sustained metabolic and glycogen phenotypes as CB1R‐linked or off‐target under our conditions. This reframing aligns with our ACEA data (transient metabolic/Ca^2+^ effects) while acknowledging ligand‐ and pathway‐bias at CB1R. Antagonist and dose–response experiments will be required to resolve receptor contributions definitively.

In this study, we present evidence suggesting the presence of CB1R on cultured astrocytes (Figure 2), consistent with previous reports (Sheng et al. 2005; Baek et al. 2008; Kofalvi et al. 2016). These results support the idea that astrocytes actively participate in cannabinoid signaling (Ramon‐Duaso et al. 2023), yet their specific role in cellular energy homeostasis remains to be fully elucidated. In this study, we found that cannabinoid receptor (CBR) signaling elevates intracellular D‐glucose, in a manner consistent with a combination of increased glucose influx and glycogen mobilization (Kofalvi et al. 2016), although we did not directly quantify transporter activity or glycogenolytic flux. We observed that ACEA, AM1241, and Gp1a stimulation increase intracellular glucose levels, yet only AM1241 and Gp1a stimulation sustain this increase over time. ACEA‐induced increase in glucose was transient, suggesting that initial glucose availability may arise from glycogenolysis. Our findings rely on two previously used CB2R ligands, AM1241 and Gp1a. Both are routinely described as selective CB2R agonists in recent work (Liu et al. 2020; Zhang et al. 2024; Cai et al. 2024; Valeriano et al. 2025). The literature reveals notable complexity in their pharmacology. AM1241 behaves as a CB2R‐preferring biased partial agonist (Soethoudt et al. 2017), displaying approximately 80‐fold greater binding affinity for CB2R (Ki ≈3.4 nM) compared to CB1R (Ki ≈280 nM) (Pertwee et al. 2010). We used the racemic mixture of AM1241 ((rac)‐AM1241). In rat CB2R, the (S)‐enantiomer has been shown to act as a CB2R agonist, although with lower potency (EC_50_ ≈785 nM), whereas the (R)‐enantiomer exhibits higher potency (EC_50_ ≈315 nM) and, in some systems, has been reported to display inverse agonism or biased signaling (Bingham et al. 2007). Gp1a shows a similarly high CB2R selectivity, yet has been reported as an inverse agonist under conditions of elevated basal activity (Soethoudt et al. 2017), contrasting with earlier descriptions of full agonism (Mussinu et al. 2003; Murineddu et al. 2006). Importantly, both ligands, AM1241 and Gp1a produced consistent phenotypic outcomes in our models. Convergent results obtained with two chemically distinct CB2‐biased agonists support the robustness of the ligand‐evoked phenotype, but, given the absence of detectable Cnr2 transcripts in our cultures, they cannot be taken as definitive evidence of CB2R mediation. ACEA is reported to be a selective agonist for CB1R, exhibiting approximately 140‐fold greater binding affinity for CB1R (Ki ≈1.4 nM) compared to CB2R (Ki ≈195 nM) (Pertwee et al. 2010).

To explore the effect of cannabinoid‐induced metabolic changes on glycogen levels, we exposed astrocytes to the CB1R‐selective agonist ACEA and the CB2‐biased ligands Gp1a and AM1241. Our findings show that application of AM1241, a CB2‐biased ligand, significantly depletes glycogen stores, in line with metabolic measurements showing sustained increases in [D‐glucose]_i_ and [L‐lactate]_i_ after 10 min of Gp1a/AM1241 stimulation. Notably, metabolic activity remained increased beyond this period, suggesting that these CB2‐biased ligands promote long‐term energy metabolism in astrocytes. In contrast, CB1R‐stimulated astrocytes exhibited no significant glycogen depletion, correlating with the rapid stabilization of [D‐glucose]_i_ and [L‐lactate]_i_ after the initial metabolic shift. By 10 min after stimulation, CB1R‐stimulated astrocytes displayed no significant metabolic differences from controls, indicating a return to baseline glycogen metabolism. However, direct comparisons between real‐time glucose fluctuations and assessments of glycogen content remain limited. We detected glucose changes within 3.3 min of acute stimulation, whereas we measured glycogen levels after 30 min, suggesting that distinct metabolic pathways are engaged over short‐ versus long‐term cannabinoid receptor activation. These findings highlight distinct metabolic profiles evoked by CB1R‐selective versus CB2‐biased ligands in astrocytes; given the lack of detectable Cnr2 transcripts in our cultures, we interpret the sustained effects of Gp1a/AM1241 as ligand‐evoked and most likely CB1‐linked and/or off‐target rather than definitive evidence of CB2R signaling. The downstream fate of glucose following CB1R activation and CB2‐biased ligand stimulation remains unresolved. Future studies, employing ^13^C‐glucose tracing through metabolic pathways, as demonstrated by Ameen et al. (2025), would be invaluable in addressing this question.

Previously, we demonstrated that intracellular Ca^2+^ signaling is a crucial activator of aerobic glycolysis in astrocytes, leading to increased [L‐lactate]_i_ and [D‐glucose]_i_ (Horvat et al. 2021). CB1R and CB2R have been reported to couple to G_q_ proteins, which activate phospholipase C, further amplifying the release of Ca^2+^ from the endoplasmic reticulum stores (Lauckner et al. 2005). In astrocytes, activation of both G_i/o_ and G_q_ pathways leads to increases in Ca^2+^, potentially driving the exocytotic release of gliotransmitters such as glutamate (Durkee et al. 2019). Consistent with this, we observed cannabinoid‐evoked Ca^2+^ rises after ACEA and after application of the CB2‐biased ligands Gp1a and AM1241; however, because Cnr2 transcripts were undetectable in our cultures, these responses are more parsimoniously interpreted as ligand‐evoked effects that are CB1‐linked and/or off‐target rather than definitive CB2R signaling. The time‐dependent nature of the Ca^2+^ responses to CB2R‐biased ligands suggests a stronger reliance on GPCR G_q/11_ activation, although spontaneous Ca^2+^ activity in astrocytes is partially independent of GPCR (Kofuji and Araque 2021b). CB1R activation likely engages both G_i/o_ and G_q_ signaling, producing a summated Ca^2+^ response from distinct pathways (Durkee et al. 2019).

Our results demonstrate that cannabinoid receptor stimulation enhances astrocytic metabolism, leading to increased [L‐lactate]_i_ and [D‐glucose]_i_ levels. CB1R activation by ACEA produced rapid but transient metabolic changes, whereas application of the CB2‐biased ligands Gp1a and AM1241 elicited sustained increases in metabolic activity. The initial metabolic response occurred within 3.3 min of receptor activation, emphasizing the rapid impact of cannabinoid signaling on astrocytic energy metabolism.

Our findings show that increases in Ca^2+^ in astrocytes occur on a similar timescale as metabolic changes, suggesting a functional coupling between cannabinoid receptor activation, Ca^2+^ signaling, and astrocytic metabolism. Acute CB1R activation by ACEA and application of the CB2‐biased ligands Gp1a and AM1241 produced distinct Ca^2+^ transients, consistent with differential engagement of intracellular signaling pathways. CB1R activation triggered rapid Ca^2+^ spikes within the first 6 min, which then declined by 10 min, aligning with previous findings (Navarrete and Araque 2008). In contrast, Gp1a/AM1241 induced a more gradual, sustained increase in Ca^2+^, peaking at the end of the 10‐min measurement period. The maximal Ca^2+^ amplitude was higher in ACEA‐stimulated astrocytes, and it exhibited a faster onset, occurring 1.4 min earlier than for Gp1a and 2.6 min earlier than for AM1241.

CB1R activation in astrocytes has been previously linked to glutamate secretion via exocytosis (Malarkey et al. 2008). Notably, CB1R‐dependent Ca^2+^ increases in astrocytes have been shown to mediate neurotransmitter release within the tripartite synapse (Navarrete and Araque 2008; Eraso‐Pichot et al. 2023). In addition, CB1R antagonism inhibited Ca^2+^ spikes triggered by 4‐aminopyridine during epileptiform activity in hippocampal slices (Coiret et al. 2012). Given that Ca^2+^ is a well‐established trigger for exocytosis in astrocytes (Kreft et al. 2004), we performed electrophysiologic measurements to confirm the direct link between CBR activation and exocytotic activity. Membrane capacitance, a proxy for exocytotic events, was increased after stimulation with all agonists used.

Our electrophysiological data did not detect significant changes in astrocytic G_m_ after stimulation with the CB2‐biased agonist Gp1a, relative to both control and ATP‐treated conditions (Figure 6D). Cannabinoid receptor activation triggers G_i/o_ protein signaling, which, via the βγ subunit, stimulates PLC (Exton 1996) and subsequently leads to depletion of phosphatidylinositol 4,5‐bisphosphate (PIP₂) from the plasma membrane. Reduced membrane PIP₂ levels have previously been implicated in the inhibition of Cx43 hemichannel opening (van Zeijl et al. 2007). Moreover, hemichannels in astrocytes can be effectively blocked by elevated intracellular Ca^2+^ (Ye et al. 2003). Patch‐clamp studies have demonstrated that a rise in cytosolic Ca^2+^ concentration leads to decreased whole‐cell permeability via hemichannel closure, potentially influencing ATP release (Stout et al. 2002). Such Ca^2+^‐mediated hemichannel blockade is considered a protective physiological mechanism (Gómez‐Hernández et al. 2003), and it could, in principle, contribute to the metabolic changes observed in this study.

While this study provides valuable insights into the roles of cannabinoid receptor signaling (predominantly CB1R, with CB2‐biased ligands acting under conditions where Cnr2 transcripts are not detectable) in astrocytic metabolism and membrane dynamics, several limitations should be acknowledged. First, although we employed two structurally distinct CB2R‐biased agonists (Gp1a and AM1241), we performed assays at a single concentration (1 μM). Full dose–response experiments or antagonist controls would further strengthen the conclusions and rule out off‐target contributions. Second, assumptions about receptor expression are constrained by the molecular data: CB1R is supported by Cnr1 transcript detection and immunocytochemistry, whereas Cnr2 transcripts were undetectable and the CB2‐like immunosignal is weak/ambiguous, so the presence of functional CB2R in these cultures cannot be confirmed. Third, we conducted Ca^2+^ imaging and membrane‐capacitance recordings in separate cell populations; thus, our data demonstrate parallel links between Ca^2+^ rises and exocytosis. Finally, experiments were limited to isolated astrocytes, leaving open question of how cannabinoid‐induced changes observed here influence neuron‐astrocyte interactions or broader neuronal network function. These limitations highlight the need for cautious interpretation and point to directions for further studies.

To summarize, our data show an association between cannabinoid‐induced Ca^2+^ rises, altered astrocytic aerobic glycolysis, and increased exocytosis in primary astrocyte cultures, predominantly under conditions where CB1R is detectable and CB2R is not.

## Author Contributions

**Katja Fink:** writing – original draft, writing – review and editing, methodology, data curation, investigation, validation. **Robert Zorec:** methodology, writing – review and editing, validation, funding acquisition. **Marko Kreft:** writing – review and editing, methodology, investigation, validation.

## Funding

This work was supported by The European Union (European Regional Development Fund, ERDF) through the Interreg VI‐A Italy–Slovenia programme, project IMMUNOCLUSTER‐2.

## Conflicts of Interest

The authors declare no conflicts of interest.

## Data Availability

The data that support the findings of this study are available from the corresponding author upon reasonable request.
